# Epidemiology with real-world data: deep endometriosis in women of reproductive age

**DOI:** 10.31744/einstein_journal/2025AO1259

**Published:** 2025-03-17

**Authors:** Nilson Abrão Szylit, Luciana Cristina Pasquini Raiza, Anucha Andrade Schindler Leal, Sérgio Podgaec

**Affiliations:** 1 Hospital Israelita Albert Einstein São Paulo SR Brazil Hospital Israelita Albert Einstein, São Paulo, SR Brazil; 2 Universidade de São Paulo Faculdade de Medicina Department of Obstetrics and Gynecology São Paulo SR Brazil Department of Obstetrics and Gynecology, Faculdade de Medicina, Universidade de São Paulo, São Paulo, SR Brazil

**Keywords:** Endometriosis, Prevalence, Pelvic pain, Diagnostic imaging, Gynecological examination

## Abstract

**Objective:**

Endometriosis is characterized by the presence of glandular tissue or endometrial stroma outside the uterus. This study aimed to determine the prevalence of symptomatic deep endometriosis and the clinical factors associated with this condition in women of reproductive age.

**Methods:**

A descriptive prevalence study combined with a cross-sectional observational study was conducted between 2017 and 2021 at a public primary care outpatient clinic in Brazil. Women of reproductive age with at least one of the following symptoms were included in the study: deep dyspareunia, dysmenorrhea, chronic pelvic pain, intestinal or urinary symptoms during the menstrual cycle, or infertility. A sample size calculation indicated that an estimated sample of 269 patients was required for the study. Qualitative variables are described as absolute and relative frequencies, while quantitative variables are expressed as medians and quartiles. Factors associated with endometriosis were analyzed using logistic regression and multiple models.

**Results:**

Data from 1,445 patients were considered. Among these, 92 were diagnosed with deep endometriosis, resulting in a prevalence of 6.4% (95%CI= 5.2-7.7) and 34.2% among women with pelvic pain (95%CI= 28.8-40.1). Women with palpable posterior cul-de-sac nodules (0R= 3.58, 95%CI= 1.68-7.63, p = 0.001) or a retroverted uterus (0R= 2.36, 95%CI= 1.28-4.37, p = 0.006) were more likely to have endometriosis.

**Conclusion:**

Using real-world data, the prevalence of deep endometriosis was found to be 6.4% in women of reproductive age and 34.2% in those with pelvic pain. The primary clinical variables associated with endometriosis are palpable posterior cul-de-sac nodules and a retroverted uterus.

## In Brief

Szylit et al. conducted a descriptive prevalence study combined with a cross-sectional observational study in a public primary care clinic. Using real-world data, they determined the prevalence of endometriosis in women selected without prior screening for the condition, employing pelvic and transvaginal ultrasound. This study represents the first of its kind in Latin America.

## Highlights

This study represents the first investigation into the prevalence of deep endometriosis within outpatient services.

Utilizing real-world data, this study explores the prevalence of endometriosis.

The prevalence of endometriosis among women of reproductive age is 6.4% (based on real-world data).

The prevalence of endometriosis in women experiencing pelvic pain is 34.2%.

Infertility is 6.5 times more common in women diagnosed with endometriosis.

The presence of palpable posterior cul-de-sac nodules or a retroverted uterus suggests endometriosis.

## Graphical abstract

**Figure:**
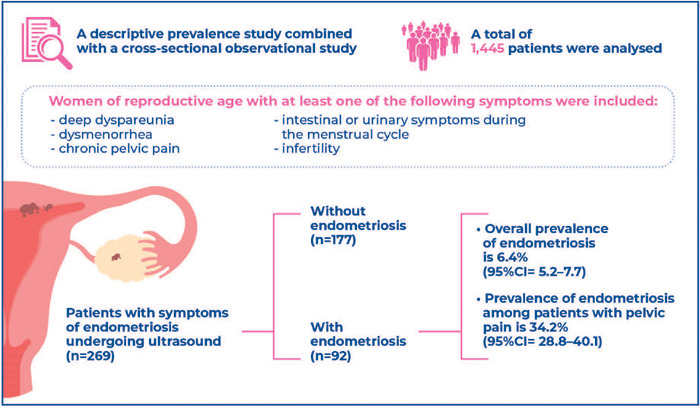

## INTRODUCTION

Endometriosis is defined as the presence of glandular epithelium or endometrial stroma outside the uterus.^(1,2)^ The primary symptoms include pelvic pain and infertility. Deep endometriosis (DE) is diagnosed when lesions extend more than 5 mm into the peritoneum.^(1)^ However, the prevalence of endometriosis has not yet been conclusively determined. Hospital-based data indicate prevalence rates ranging from 4% to 50%. In contrast, population-based studies have estimated rates between 0.3% and 4.8%^(3,4,5,6,7,8)^ which increases to 10% in women of reproductive age.^(2,7)^

Currently, imaging methods, such as transvaginal pelvic ultrasound (TVPUS) and magnetic resonance imaging, are recommended for their ability to identify ovarian endometriomas and DE with high accuracy, achieving detection rates comparable to laparoscopy.^(9,10,11,12)^ Transvaginal pelvic ultrasound has a lower cost and greater availability, thus avoiding diagnostic delays.^(1)^ Understanding the true prevalence of endometriosis in the general population is the key to identifying individuals who may have the disease, directing healthcare resources, and effectively managing the affected population.

To the best of our knowledge, this study is the first to investigate the prevalence of symptomatic DE in a population treated with spontaneous and sequential demands in an outpatient health service.

## OBJECTIVE

This study aimed to determine the prevalence of severe symptomatic deep endometriosis and the clinical factors associated with this condition in women of reproductive age.

## METHODS

### Study design, data source and sample population

A descriptive prevalence study combined with a cross-sectional observational study was conducted between June 22, 2017, and March 16, 2021, in a public municipal primary care health unit in São Paulo. The study adhered to the Strengthening the Reporting of Observational Studies in Epidemiology (STROBE) checklist.^(13)^ The study was approved by the *Hospital Israelita Albert Einstein* and the Municipal Health Department Research Ethics Committee (CAAE: 61310116.4.0000.0071; # 1.835.198) and the *Secretaria Municipal da Saúde* (CAAE: 61310116.4.3001.0086; #1.882.196). All participants provided informed consent or informed assent when appropriate. The sample size was estimated for a finite population, assuming a 2% prevalence of endometriosis^(14)^ and a population of approximately 7,000 women of reproductive age receiving care at the facility. The sample size was calculated using a 95% confidence interval (95% CI) and an absolute precision of 2.6%, resulting in a total of 269 participants.

The inclusion criteria were individuals aged ≥15 years and up to menopause, along with any of the following symptoms: deep dyspareunia (DD), dysmenorrhea, chronic pelvic pain (CPP), intestinal or urinary symptoms during the menstrual cycle, or infertility. The exclusion criteria were the inability to answer the questionnaires; pregnancy; an intact hymen; ovarian insufficiency (defined as amenorrhea lasting longer than three months accompanied by elevated serum follicle-stimulating hormone levels greater than 25 IU/L in two measurements taken at least 30 days apart); or the presence of fibroids >4cm as identified via previous vaginal ultrasonography. This criterion was established due to the high prevalence of leiomyomas in the population, the positive correlation between leiomyoma size and the intensity of dysmenorrhea reported by patients, and their potential to confound the diagnosis of endometriosis.^(15)^ All participants, who were seen consecutively on demand, were asked to complete a structured screening questionnaire regarding the aforementioned symptoms. The questionnaires were submitted to a single gynecologist who selected the participants based on the inclusion and exclusion criteria. Gynecological appointments included the collection of demographic data, information on comorbidities and contraceptive methods, menstrual and obstetric histories, and the presence and intensity of symptoms. Pain was assessed using a visual analog scale (VAS) ranging from 0 to 10.

All participants who reported a score of ≥7 for any pain symptom, the presence of menstruation-related urinary or intestinal symptoms, or infertility without a diagnosed cause were referred for physical examination and TVPUS for the mapping of endometriosis. The criteria included participants experiencing moderate-to-high pain (scores up to 7) and severe pain (scores above 8), both of which significantly affect physical and mental health. Pain levels are also considered indicators of clinical treatment failure, suggesting the need for surgical intervention. This criterion has been used in similar studies.^(16,17,18)^

The physical examination included abdominal palpation, bimanual vaginal examination, and speculum examination. The presence of thickening or nodules in the retrocervical region, uterosacral ligaments, posterior vaginal wall, or lateral, anterior, or posterior vaginal fornices during the vaginal examination was considered indicative of suspected endometriosis. We assessed uterine volume, position, mobility, and pain resulting from manipulation during the examination.

All patients who attended the consultation underwent a gynecological examination and were referred for TVPUS regardless of the findings from the physical examination.

Endometriosis was diagnosed based on the presence of lesions on TVPUS, using a specific protocol for mapping the disease in accordance with the recommendations of the Deep Endometriosis Analysis consensus. This protocol includes an assessment of the uterus, ovaries, mobility of pelvic structures, tender points, and both anterior and posterior pelvic compartments.^(19)^ All tests were conducted by a single radiologist with over 10 years of experience who was blinded to the clinical findings. All ultrasound examinations were performed by a single radiologist with over 10 years of experience, who was blinded to the clinical findings. The ultrasounds were conducted within an interval of up to 3 months after the clinical evaluation. An Epiq 7 Ultrasound System (Philips Bothell Washington) was used with convex (C 5-1, 1-5 MHz), endocavitary (C 10-3v, 3-10 MHz), and linear (L3-12, 3-12 MHz and L5-12, 5-12 MHz) probes.

To calculate the proportion of endometriosis cases, we considered women who were initially screened without suspicious criteria alongside those who underwent TVPUS in the denominator. In the numerator, we included patients with a confirmed diagnosis of endometriosis based on TVPUS findings.

Qualitative data are described using absolute frequencies and percentages, while quantitative data are presented as means and standard deviations or medians and quartiles, along with minimum and maximum values. Missing data were not imputed, and the analysis was conducted using the available information. Factors associated with the presence of endometriosis were analyzed using logistic regression models employing both simple and multiple approaches, with results presented as odds ratios (ORs), 95%CIs, and significance probabilities (p-values). The final multiple model was obtained after variable selection through likelihood ratio tests, ensuring that only significant variables at the 5% level remained in the model. Subgroup or interaction effects were not investigated in the multiple models. IBM SPSS Statistics for Windows software (version 26.0; IBM, Armonk, NY, USA) was used for all analyses, with a significance level set at 0.05.

## RESULTS

**Figure 1:**
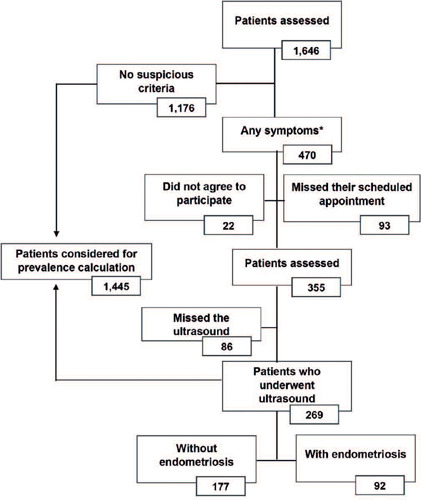
Flowchart of patient selection for the study

Figure 1 illustrates the participant selection process. Initially, 1,646 women responded to the first questionnaire. Of these, 1,176 did not meet the inclusion criteria. The remaining 470 patients were referred for consultation and physical examination, while 93 missed their scheduled appointments. Of the 377 patients who attended the consultation, 22 did not agree to participate in the study, and 86 agreed but missed their scheduled ultrasound. Consequently, data from 1,445 patients were considered for the calculation of DE prevalence, consisting of 1,176 patients without signs and symptoms characteristic of endometriosis (VAS <7) and 269 patients who agreed to participate in the study and underwent TVPUS. Among these, 92 were diagnosed with DE, resulting in a prevalence of 6.4% (95%CI= 5.2-7.7) in the overall study population.

* Any of the following symptoms: dysmenorrhea, deep dyspareunia, chronic pelvic pain, cyclic intestinal or urinary symptoms, or infertility

Among participants with suspected endometriosis who underwent TVPUS, the prevalence was 34.2% (95%CI = 28.8-40.1).

The symptoms used to select the participants and their frequencies in each subgroup are listed in table 1. Infertility was reported 6.5 times more frequently in women diagnosed with endometriosis compared to the 1,445 patients considered in the calculation of endometriosis prevalence.

**Table 1:** Frequency of endometriosis-related symptoms (using the Visual Analog Scale) and signs

Symptoms and signs	Total number of participants (n=1,646)	Underwent TVPUS (n=269)	Underwent TVPUS with endometriosis (n=92)
Dysmenorrhea ≥ 7&	388 (23.6)	214 (79.6)	78 (84.8)
Deep dyspareunia ≥ 7&	213 (12.9)	120 (44.6)	41 (44.6)
Chronic pelvic pain ≥ 7&	139 (8.4)	74 (27.5)	25 (27.2)
Intestinal disorders	175 (10.6)	94 (34.9)	37 (40.2)
Urinary disorders	94 (5.7)	61 (22.7)	17 (18.5)
Infertility	47 (2.9)	34 (12.6)	15 (16.3)

& Buy a visual analog scale.

The demographic data of 269 women who underwent ultrasonography were analyzed. The variables were similar between the participants with and without endometriosis, indicating that the two groups were homogeneous. Regarding contraceptive methods, 13.4% of the participants reported not using any contraceptives, 35.3% used hormonal pills, 18.2% used condoms, 12.3% had undergone tubal ligation, 7.8% had a vasectomy, 2.2% used a copper intrauterine device, 1.9% practiced coitus interruptus, 0.7% had a hysterectomy, and 0.7% used other methods. Five (1.9%) participants reported no sexual activity.

The mean age of the participants without endometriosis who underwent TVPUS and those with endometriosis was 33 years, with standard deviations of 8 and 7 years, respectively. The racial distribution among participants was homogeneous. Of the 177 participants without endometriosis, 23 (13.0%) identified as Black, 99 (55.9%) as Pardo, 54 (30.5%) as White, and one (0.6%) as Asian. Among those with endometriosis, 10 (10.9%) identified as Black, 49 (53.3%) as Pardo, and 33 (35.9%) as White. The median body mass indices of the participants with and without endometriosis were 25 and 26 kg/m^2^, with first and third quartiles of 23 and 30, and 23 and 29, respectively.

There was no relationship between menstrual flow characteristics and endometriosis. Participants who experienced menarche after the age of 13 years did not have a higher probability of developing endometriosis compared to those who experienced menarche before the age of 12 years (OR= 1.49, 95%CI = 0.75-2.96). The presence of endometriosis was not associated with either large or small menstrual volume (OR= 1.67, 95%CI= 0.77-3.66); short (<25 days), or long (>35 days) menstrual cycles (OR= 1.2, 95%CI= 0.32-4.50); or last menstrual flow (<5 or >7 days) (OR= 1.09, 95%CI= 0.32-3.29).

Obstetric history did not influence the incidence of endometriosis. Participants with a history of pregnancy (OR= 0.77, 95%CI= 0.44-1.32), cesarean section (OR= 1.19, 95%CI= 0.70-2.03), vaginal delivery (OR= 0.71, 95%CI= 0.43-1.18), or abortion (OR= 1.39, 95%CI= 0.76-2.57) did not exhibit increased rates of endometriosis.

The proportion of reported endometriosis symptoms was similar between participants with and without endometriosis who underwent TVPUS (Table 2). Endometriosis suspected during physical examination was confirmed using TVPUS in 55 patients (59.8%). Most suspected cases were identified through bimanual examination (98.2% in the endometriosis group), while speculum examination detected only one case.

**Table 2:** Comparison of symptoms characteristic of endometriosis in patients with and without endometriosis undergoing transvaginal pelvic ultrasound

	Total (n=269)	Endometriosis	
		No (n=177)	Yes (n=92)
Dysmenorrhea#	214 (79.6)	136 (76.8)	78 (84.8)
Deep dyspareunia#	120 (44.6)	79 (44.6)	41 (44.6)
Chronic pelvic pain#	74 (27.5)	49 (27.7)	25 (27.2)
Intestinal symptoms during menstruation	94 (34.9)	57 (32.2)	37 (40.2)
Urinary symptoms during menstruation	61 (22.7)	44 (24.9)	17 (18.5)
Infertility	34 (12.6)	19 (10.7)	15 (16.3)

# Type of pain with intensity ≥7 (measured by a Visual Analog Scale).

Regarding the position of the uterus in the group without endometriosis, 77.4% were anteverted, 16.4% were retroverted, 5.6% were in a neutral position, and one was absent. In the endometriosis group, 58.7% were anteverted, 34.8% were retroverted, 5.4% were in a neutral position, and one was absent. Retrocervical thickening was observed in 26.6% of participants without endometriosis and in 43.5% of those with endometriosis. The proportions of nodules palpated in the retrocervical region were 7.3% and 25% in the groups with and without endometriosis, respectively.

An adnexal mass was present in 7.9% and 5.4% of patients, and posterior cul-de-sac (PCDS) bulging was observed in 4.0% and 7.6% of participants with and without endometriosis, respectively.

Transvaginal pelvic ultrasonography revealed a median uterine volume of 80cm^3^ (first and second quartiles: 60cm^3^ and 105cm^3^) in participants without endometriosis. In contrast, participants with endometriosis had a median uterine volume of 90 cm^3^ (first and second quartiles: 60cm^3^ and 115cm^3^), with no significant difference between the groups (p=0.098).

Among the 92 women diagnosed with endometriosis, the number of lesions identified via TVPUS ranged from one to four distinct lesions per individual. The most frequently observed lesion was retrocervical endometriosis, present in 88 women (95.7% of the endometriosis cohort), followed by endometriosis in the upper third of the vagina (19 cases, 20.7%), ovarian endometrioma (10 cases, 10.9%), rectosigmoid endometriosis (8 cases, 8.7%), bladder endometriosis (3 cases, 3.3%), and appendix endometriosis (1 cases, 1.1%).

Simple logistic regression models, which considered the participants' clinical conditions and physical examination findings, demonstrated an association between the suspected gynecological findings and imaging diagnoses. During the gynecological examination, participants with palpable PCDS nodules and a retroverted uterus were more likely to be diagnosed with endometriosis (Table 3).

**Table 3:** Association between symptoms and risk signs for endometriosis, vaginal examination findings, and endometriosis diagnosis by ultrasound using simple logistic regression models

	Simple logistic regression models	
	OR (95%CI)	p value
Signs and symptoms		
Dyspareunia	1.26 (0.74:2.12)	0.394
Dysmenorrhea	1.72 (0.66:4.48)	0.264
Intestinal symptoms during menstruation	1.42 (0.84:2.39)	0.192
Urinary symptoms during menstruation	0.69 (0.37:1.28)	0.237
CPP	0.95 (0.57:1.58)	0.852
Infertility	1.62 (0.78:3.36)	0.195
Physical examination		
Gynecological examination suspicious of endometriosis	2.22 (1.33:3.71)	0.002
Retro-cervical thickening	2.13 (1.25:3.62)	0.005
PCDS nodules	4.21 (2.01:8.78)	<0.001
Adnexal mass	0.67 (0.23:1.92)	0.455
Pain during cervical manipulation	0.79 (0.46:1.35)	0.390
Mobile uterus	0.89 (0.45:1.74)	0.727
Uterus position*		
Retroverted	2.80 (1.55:5.07)	0.001
Neutral	1.27 (0.41:3.88)	0.677

* The anteflexed uterine position was used as the reference for calculating odds ratios using regression analysis.

OR: odds ratio; 95%CI: 95% confidence interval; CPP: chronic pelvic pain; PCDS: posterior cul-de-sac.

In a multivariate model adjusted for the variables of interest following significant variable selection, the presence of palpable PCDS nodules and a retroverted uterus was associated with the diagnosis of endometriosis as determined by TVPUS (Table 4). The significant variables in the multiple models aligned with those in the simple models.

**Table 4:** Association between symptoms and risk signs for endometriosis, vaginal examination findings, and endometriosis diagnosis by ultrasound using regression multiple logistic models

	Multiple logistic regression models	
	OR (95%CI)	p value
Physical examination		
PCDS nodules	3.58 (1.68:7.63)	0.001
Uterus position*		
Retroverted	2.36 (1.28:4.37)	0.006
Neutral position	1.21 (0.39:3.81)	0.744

* The anteflexed uterine position was used as the reference for calculating odds ratios using regression analysis.

PCDS: posterior cul-de-sac; OR: odds ratio; 95%CI: confidence interval

## DISCUSSION

Using real-world data, this study demonstrated that the prevalence of symptomatic endometriosis diagnosed by TVPUS was 6.4% in a community in São Paulo, Brazil.

The estimated prevalence of endometriosis is 10%.^(2)^ However, some studies have indicated that this percentage pertains to populations with surgical indications, therefore introducing a significant selection bias.^(9)^ Conversely, studies based on hospital records have reported very low prevalence rates.^(4)^ The high clinical heterogeneity of women selected for endometriosis studies partly explains the different prevalence rates reported in the literature.^(9)^

Zondervan et al.^(2)^ called for more conclusive epidemiological and clinical data obtained through noninvasive diagnostic methods, as current knowledge is biased due to the difficulties faced by the population in accessing healthcare services.

In a 2020 meta-analysis of 28,660,652 women, Sarria-Santamera et al. found a prevalence rate of 1%-5%. High heterogeneity among the studies was reported based on statistical calculations of I^2^. The authors concluded that this heterogeneity was attributable to variability in case definitions, subject selection strategies, and inherent variability in endometriosis. The pooled analysis showed that the prevalence of self-reported endometriosis was 5% (95%CI= 0.03-0.06).^(9)^

The clinical assessment of endometriosis relies on symptoms that, while characteristic, are also present in conditions such as irritable bowel syndrome, painful bladder syndrome, and complications of pelvic inflammatory disease.^(2)^

In this study, the majority of the demographic and clinical variables examined were not associated with endometriosis, making it impossible to conduct a multivariate analysis that included clinical data and history. It is important to emphasize that the study design involved screening patients with suspected endometriosis who were diagnosed using TVPUS; therefore, all participants presented with pelvic pain, which served as a limiting factor for this analysis.

Among the participants with pelvic pain who underwent TVPUS, 34.2% were found to have endometriosis, which falls within the range reported by the American College of Obstetricians and Gynecologists, indicating an endometriosis prevalence of 70% (range, 28-93%; 95%CI= 67-73).^(20)^

In a systematic review involving 459,975 participants, a meta-regression analysis indicated dysmenorrhea rates ranging from 16.8% to 81%, DD rates between 8% and 21.8%, and CPP rates from 2.1% to 24%.^(21)^ These findings are consistent with the prevalence of endometriosis calculated from the data of 1,445 patients in the present study.

In Brazil, a retrospective study evaluated 1,116 participants who were surgically diagnosed with endometriosis and reported rates of dysmenorrhea, DD, CPP, and infertility of 76.5%, 44.2%, 36.6%, and 41.9%, respectively.^(16)^ In the present study, we observed a higher rate of dysmenorrhea, a similar rate of DD, and a lower rate of CPP in participants diagnosed with endometriosis using TVPUS.

This difference may be attributed to the prospective design of our study, in which participants were interviewed in person prior to any clinical or surgical treatment, which improved data accuracy.

More precise prevalence estimates should account for the study population, reasons for appointments, patient referrals, and sociodemographic factors.^(22)^

In this study, the infertility rate in women with endometriosis was lower than the 30%-50% reported in the literature.^(8,23)^ However, more than half of the participants who underwent TVPUS did not express a desire for children. We highlight that the initial participants were selected spontaneously, with no active search for cases of infertility. It is essential to consider the possibility that women experiencing infertility may require specialized reproductive services.

The diagnosis of endometriosis through vaginal examination presents significant challenges. Palpable nodules or thickening posterior to the cervix may be related to nearby structures, making it difficult to ascertain the exact location or extent of the lesions.^(10)^

In this study, all participants undergoing TVPUS experienced pelvic pain; therefore, the findings from the vaginal examination findings may be related to other etiologies, such as adhesions resulting from pelvic infectious processes or increased ovarian volume, which complicate diagnosis by vaginal examination alone.^(24)^ Previous data have shown a tendency toward a positive association between a retroverted uterus and endometriosis. Although this finding may be observed in 15-20% of women, which falls within the normal range, DE can lead to adhesions, favoring a retroverted position.^(25)^

A recent meta-analysis involving 4,565 participants reported a sensitivity of 71% and a specificity of 69%, demonstrating adequate diagnostic accuracy (area under the curve = 0.76) for endometriosis based on physical examination.^(26)^

The results of previous studies involving patients requiring surgical treatment cannot be directly compared to those of the present study.

This study has some limitations. Only participants experiencing pelvic pain completed the second questionnaire and underwent a physical examination and TVPUS, preventing the comparison of sociodemographic data between women with and without endometriosis symptoms. The high rate of contraceptive use in this sample may have diminished both the infertility rate and reported pain levels.

Additionally, endometriosis was diagnosed solely in patients exhibiting symptomatic disease and moderate-to-severe pain. The exclusion of women with leiomyomas greater than 4cm, a condition associated with endometriosis, may have introduced a selection bias. This criterion was adopted to limit the screening of cases referred for TVUS and because the pain reported by the patient was related to the presence of the myoma.^(15)^

Uterine fibroids affect approximately 70% of women aged 35 to 49 years, with an even higher incidence (80%) among Black women in the United States.^(27)^ This condition shares many symptoms with endometriosis, making differential diagnosis challenging without imaging studies.^(28)^

Another review reported a rate of uterine fibroids of 20 to 25% in women of reproductive age, and 30 to 40% in women older than 40 years.^(29)^ This overlap could have led to an overrepresentation of fibroid cases and, consequently, an underestimation of the prevalence of endometriosis in the study, as patient referrals for TVPUS were often driven by symptoms more commonly associated with fibroids.

Including all women diagnosed with fibroids would have required a substantial increase in the number of TVPUS, making the study logistically unfeasible.

Importantly, these patients were already receiving appropriate clinical management, and their need for surgical planning would not have been influenced by the prevalence rates established within the general population, as is typical in real-world scenarios.

Similarly, most patients experiencing severe pain are not treated according to clinical protocols aimed at the diagnosis and management of pain. Lastly, we were unable to prospectively follow patients with endometriosis who were referred for surgery due to logistical limitations.

The strengths of our study included the inclusion of single-center participants assessed by a single gynecologist and ultrasonography evaluations conducted by a single radiologist with experience in this area. The data were obtained prospectively, thereby reducing the risk of data loss or recall bias and simulating real-world conditions without specific screening or reliance on data from specialized centers for pelvic pain, infertility, or endometriosis.^(9)^

This was the first national epidemiological study to determine the prevalence of endometriosis. Therefore, similar studies using other population samples are essential due to the multifactorial nature of endometriosis.

The prevalence of endometriosis was 6.4%, which is lower than that reported in the literature,^(2)^ highlighting the need for appropriate equipment and adequately trained personnel for the diagnosis and treatment of this condition.

## CONCLUSION

The prevalence of deep endometriosis in this population was lower than that reported in the literature, yet it was higher among women with a clinical suspicion of endometriosis who were subsequently diagnosed using transvaginal pelvic ultrasound. Participant selection based on symptoms and findings from physical examination is essential for justifying referrals for imaging tests. This approach leads to an accurate diagnosis regarding the potential presence and extent of endometriosis and ensures that the necessary material and human resources, equipped with proper training, are available for effective population health care.
